# Impact of altered vena cava flow rates on right atrium flow characteristics

**DOI:** 10.1152/japplphysiol.00649.2021

**Published:** 2022-03-10

**Authors:** Louis P. Parker, Anders Svensson Marcial, Torkel B. Brismar, Lars Mikael Broman, Lisa Prahl Wittberg

**Affiliations:** ^1^FLOW and BioMEx, Department of Engineering Mechanics, Royal Institute of Technology, KTH, Stockholm, Sweden; ^2^Division of Medical Imaging and Technology, Department of Clinical Science, Intervention and Technology, Karolinska Institutet, Stockholm, Sweden; ^3^Department of Radiology, Karolinska University Hospital in Huddinge, Stockholm, Sweden; ^4^ECMO Centre Karolinska, Pediatric Perioperative Medicine and Intensive Care, Karolinska University Hospital, Stockholm, Sweden; ^5^Department of Physiology and Pharmacology, Karolinska Institutet, Stockholm, Sweden

**Keywords:** basic science research, computerized tomography (CT), hemodynamics, right atrium, vena cava

## Abstract

The right atrium (RA) combines the superior vena cava (SVC) and inferior vena cava (IVC) flows. Treatments like extracorporeal membrane oxygenation (ECMO) and hemodialysis by catheter alter IVC/SVC flows. Here we assess how altered IVC/SVC flow contributions impact RA flow. Four healthy volunteers were imaged with computerized tomography (CT), reconstructed and combined into a patient-averaged model. Large eddy simulations (LESs) were performed for a range of IVC/SVC flow contributions (30%–70% each, increments of 5%) and common flow metrics were recorded. Model sensitivity to reconstruction domain extent, constant/pulsatile inlets, and hematocrit was also assessed. Consistent with literature, a single vortex occupied the central RA across all flowrates with a smaller counter-rotating vortex, not previously reported, in the auricle. Vena cava flow was highly helical. RA turbulent kinetic energy (TKE; *P* = 0.027) and time-averaged wall shear stress (WSS; *P* < 0.001) increased with SVC flow. WSS was lower in the auricle (2 Pa, *P* < 0.001). WSS in the vena cava was equal at IVC/SVC = 65/35%. The model was highly sensitive to the reconstruction domain with cropped geometries lacking helicity in the venae cavae, altering the RA flow. The RA flow was not significantly affected by constant inlets or hematocrit. The commonly reported vortex in in the central RA is confirmed; however, a new, smaller vortex was also recorded in the auricle. When IVC flow dominates, as is normal, TKE in the RA is reduced and WSS in the venae cavae equalize. Significant helicity exists in the vena cava, as a result of distal geometry and this geometry appears crucial to accurately simulating RA flow.

**NEW & NOTEWORTHY** Right atrium turbulent kinetic energy increases as the proportion of flow entering from the superior vena cava is increased. Although the commonly reported large right atrium vortex was confirmed across all flow scenarios, a new smaller vortex is observed in the right auricle. The caval veins exhibit highly helical flow and this appears to be the result of distal venous morphology.

## Graphical abstract

**Figure:**
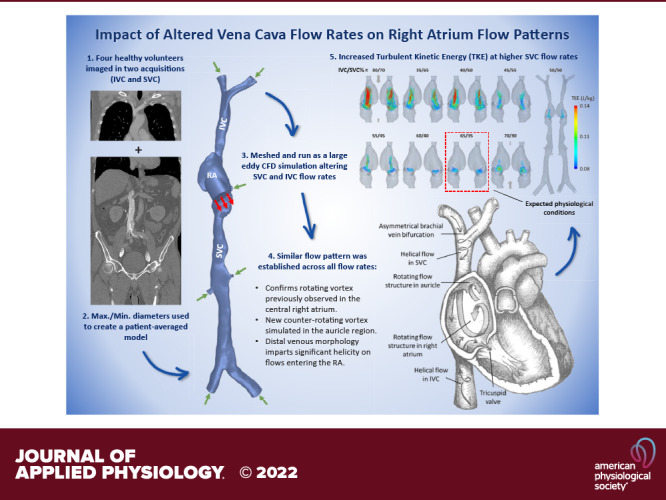

## INTRODUCTION

Extracorporeal membrane oxygenation (ECMO) is a treatment used in severe cardiac and/or respiratory failure. Its demand has increased during the global coronavirus (COVID-19) pandemic (1). In veno-venous (VV) ECMO, used for respiratory support, blood is drained from the inferior vena cava (IVC) and oxygenated blood is returned to the superior vena cava (SVC), or vice versa. Alternatively, a dual-lumen cannula can be applied. Under ECMO, the natural balance of flows entering the right atrium (RA) is altered. For cardiopulmonary support, veno-arterial ECMO may be offered where drainage is performed from a caval vein and/or the RA. Much like ECMO, hemodialysis via a central dialysis catheter requires the drainage and return of venous blood through a cannula that is usually placed in the SVC. In addition to these, caval flow rates might also be affected by pregnancy (2), thrombosis (3), or compression by tumor (4), referred to, depending on location, as SVC or IVC syndrome.

The flow characteristics of the left side of the heart and the right ventricle are well described in literature although the RA has received little attention (5). The few reports to date consist largely of qualitative descriptions of flow, based on four-dimensional magnetic resonance imaging (4-D-MRI). These note a common pattern of a single forward turning vortical structure in the RA (5, 6), theorizing that the rotational nature of the flow retains kinetic energy, directing flow toward the tricuspid valve and reducing the work required by the RA wall (5). Computational fluid dynamics (CFD) offers similar flow data to 4-D-MRI and has been used to assess the flow in the vena cava (7, 8), cardiac ventricles (9) but not the complete IVC, SVC, and RA anatomy together. CFD analysis offers a more detailed velocity field compared with 4-D-MRI, though the two are complementary means to assess flow. Four-dimensional magnetic resonance imaging allows for direct observation of in vivo hemodynamics, whereas CFD enables assessment of the hemodynamics through detailed simulations. Both methodologies are associated with sensitivities that are important to quantify.

Large eddy simulation (LES) is a numerical method whereby large eddies in the flow are directly resolved on the computational mesh. The effect of smaller eddies, below the grid size, are then accounted for by a subgrid scale (SGS) model. Some degree of turbulence might reasonably be expected in the RA where IVC and SVC flow meet nearly head-on and disturbed flow has previously been reported in imaging (5, 10). LES performs well in transitional flows and has been used previously to analyze the emergence of turbulence in the arteries (11, 12).

In this study, we aim to understand the effect of altered caval flow contributions on the underlying RA flow characteristics, providing insight into how ECMO, hemodialysis by central venous catheter, and IVC/SVC syndrome might impact RA function.

## METHODS

### Imaging and 3-D Reconstruction

Four healthy volunteers (3 females, 1 male, age: 58.3 ± 3.5 yr) gave informed consent and were imaged at the Department of Radiology at Karolinska University Hospital, Huddinge, Sweden following institutional guidelines. A dual-source multidetector Somatom Definition Flash (Siemens Healthcare, Forchheim, Germany) was used with a slice thickness of 0.75 mm. Two image acquisitions were made with intravenous injection of a solution consisting of iodinated contrast media and saline injected simultaneously in *1*) right and left foot and *2*) right and left arm, capturing the SVC, IVC, and RA, gated for systole. To reduce the effect of breathing on vein morphology, all images were acquired during inspiratory breath-hold. To assess motion in the SVC, IVC, and RA, and how this might affect our analysis, additional phase-contrast computerized tomography (CT) imaging of five unrelated subjects was obtained with a Revolution CT (GE Healthcare, Milwaukee, WI) at a slice thickness of 0.625 mm. The gated imaging domain covered the RA and ∼5 cm of the IVC and SVC from which diameter measurements could be derived. All subjects gave informed consent and ethics approval was obtained from the Swedish Ethical Review Authority (Ethical Permit No. 2018/438-31).

**Figure 1.**
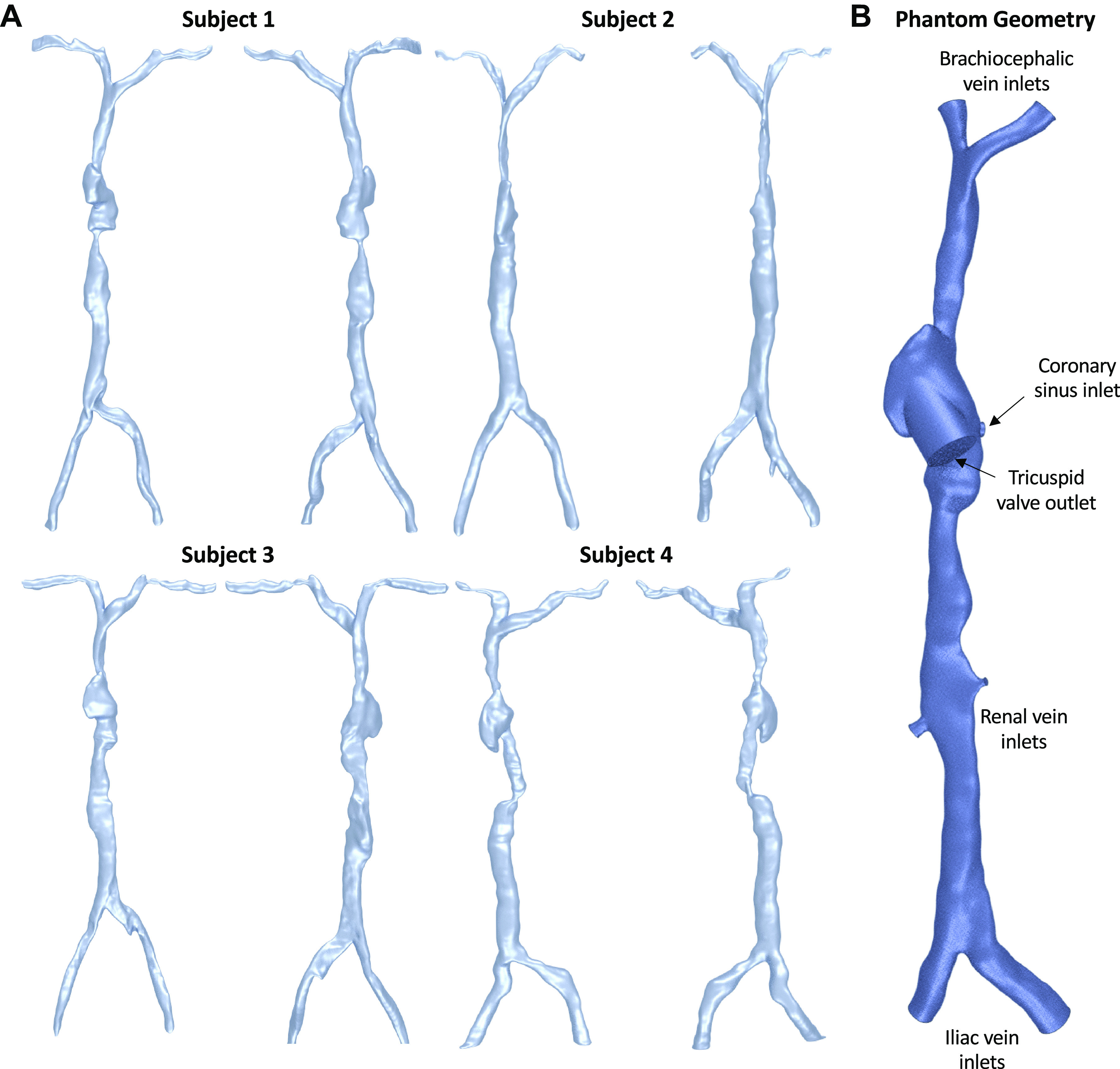
*A*: three-dimensional (3-D) reconstructions of the four healthy subjects imaged. Anterior (*left*) and posterior (*right*) shown for each subject. *B*: the phantom geometry (anterior view) used for computational fluid dynamics (CFD) analysis.

For the static imaging, the two separate acquisitions (abdomen and chest) for each subject were merged. The caval veins and RA were reconstructed into three-dimensional (3D) by Hounsfield value using the software package 3D Slicer (v. 4.10.2) by a single analyst. Reconstructions were made from the brachial to iliac veins (Fig. 1*A*). Removal of imaging artifacts by manual editing and smoothing was performed in STAR-CCM+ (Siemens, Munich, Germany) using methods previously reported (13). Minimum and maximum diameter, centerline length, and centerline tortuosity were measured for all major veins. In addition to these, major and minor axis length (in axial CT), volume, and surface area were recorded for the RA. These measurements were used to establish mean values for maximum and minimum vein diameters and the dimensions of the RA. Individual vein segments were then selected, scaled, and merged to create a patient-averaged phantom model that best reflected mean values (Fig. 1*B*; Supplemental Table S1; all Supplemental material is available at https://doi.org/10.6084/m9.figshare.16602209). Although the location and orientation of the tricuspid valve and coronary sinus were visible in the imaging data, exact diameters could not be quantified. Assuming a circular shape, the coronary sinus was given a diameter of 0.7 cm (14) and tricuspid valve was 2.5 cm (15), reflecting population averages reported in literature. For correct orientation of the tricuspid valve, a cylindrical extrusion into the RA was made (Fig. 1*B*). For the transient imaging data, minimum and maximum diameters of the SVC, IVC, and RA were measured at six equally spaced time points in the cardiac cycle using 3D Slicer.

### Flow Analysis

CFD simulations of the phantom model were performed in STAR-CCM+. k-ɛ Reynolds-averaged Navier–Stokes (RANS) was used to initialize the flow field. This was followed by a large eddy simulation (LES) with a wall-adapting local eddy-viscosity (WALE) SGS model. A more recent formulation of the Smagorinsky model (16), the WALE model (17) is similarly implemented through a turbulent viscosity:

(*1*) $$\mu_{t}=\;\rho \Delta^{2}S_{W},$$

where *ρ* is density, Δ is the length scale, and *S_W_* is a deformation parameter (defined in Supplemental Material) (18). The length scale limit was set as follows:

(*2*) $$\Delta \;=\;\min\;(kd,\;C_{w}V^{\frac{1}{3}}),$$

where *k* is the von Karman constant (= 0.41), *d* is the wall distance, and *C_W_* is the WALE model coefficient (= 0.544) and *V* is cell volume (18).

The WALE performs well in complex, wall-bounded geometries and hence its implementation in the present study. A constant time step of 10^−4^ s was used with second-order temporal discretization. Mesh independence was assessed on a cropped model centered on the RA, the primary region of interest, with three meshes of increasing density (2.5, 8.8, and 20.2 M cells). A mesh independence study was conducted giving a grid convergence index (GCI) <3% for velocity in the RA (Supplemental Fig. S1 and Table S2) on the medium density mesh. Time-averaged wall shear stress (TAWSS) and turbulent kinetic energy (TKE) were also computed for each of these meshes showing good agreement between the medium and fine mesh results (Supplemental Fig. S2 and Table S3). When applied to the entire geometry, these meshing parameters resulted in an 11.2 M cell mesh (mean cell size = 0.29 mm). The final mesh comprised polyhedral core cells with 16 prism layers and inlet/outlet extensions >10 times the local vessel diameter. A rigid-wall model was implemented with a constant total inflow of 6 L/min, distributed to inlets by surface area [excluding the coronary sinus, which was allocated 2% of the total flow (19) in all simulations]. The tricuspid valve was set as a zero-pressure outlet. The proportion of flow from the IVC/SVC% was simulated at 30/70%, 35/65 %, 40/60%, 45/55%, 50/50%, 55/45%, 60/40%, 65/35%, and 70/30%, where 65/35% reflects a previously reported average for healthy adults (20). Blood density of 1,050 kg/m³ and a Quemada viscosity model (21) were implemented to account for the shear-thinning behavior of blood. A hematocrit of 35% was used in the Quemada model to reflect a typical patient on ECMO, where hematocrit is typically in the range of (20%–40%) (22, 23) giving a volume-average viscosity of 0.0030 Pa.s for the 50/50 flow scenario. Simulations were run for 1.5 s of physical time with momentum and continuity residuals converging to <10^−5^. Velocity and wall shear results were averaged over the final 1 s. This was run on 500 cores of the Kebnekaise Supercomputer (High Performance Computing Center North, Umeå, Sweden), each simulation requiring ∼20 h to complete.

**Figure 2.**
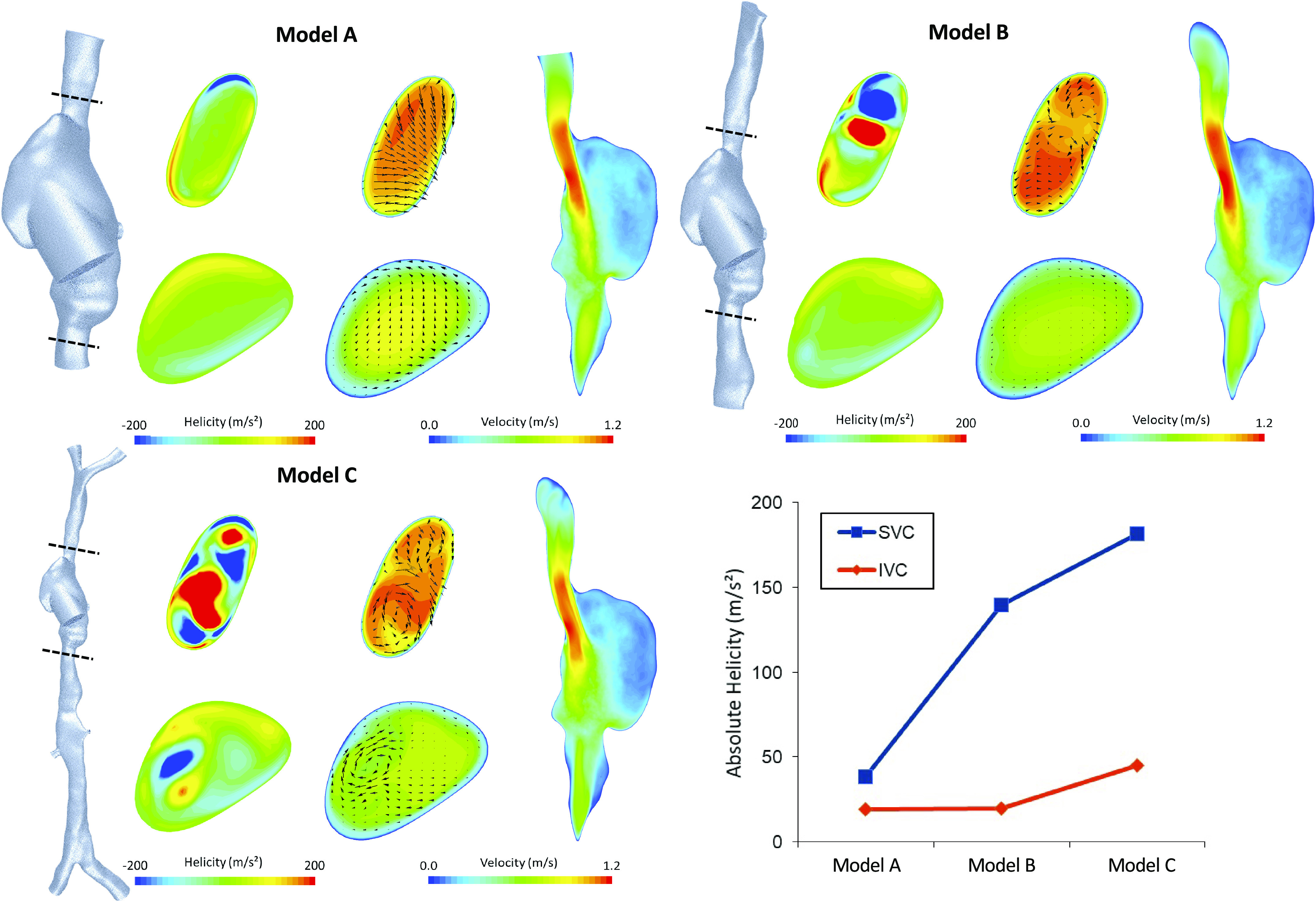
Helicity and velocity in axial planes through the superior vena cava (SVC) and inferior vena cava (IVC) and velocity at a cross-sectional plane through the right atrium for *Models A*, *B,* and *C*. All results shown are for the scenario where SVC and IVC flows are equal. Surface-averaged helicity on the axial planes through the SVC and IVC is plotted for the three models (*bottom right*).

The sensitivity of velocity results to several modeling assumptions was assessed. To determine the geometric complexity required for accurate results in the RA, three models of differing complexity (*Models A*, *B*, and *C*, Fig. 2) were compared for equal caval flows. Transient inlet conditions (24), representing pulsatile venous flow, were compared with constant conditions. The IVC and SVC waveforms applied had pulsatility indexes of 2.45 and 2.29, respectively, and are plotted in the Supplemental Fig. S3. The sensitivity of the model to a range of hematocrit values (25%–50%) was also quantified (see Supplemental Fig. S4).

Turbulent kinetic energy (TKE) was calculated as the sum of resolved TKE (Eq. *3*) and subgrid-scale TKE (25) (Eq. *4*).

(*3*) $$k_{r}=\frac{1}{2}\left(\;\bar{{\left(u^{\prime }\right)}^{2}}+\;\bar{{\left(v^{\prime }\right)}^{2}}+\;\bar{{\left(w^{\prime }\right)}^{2}}\right)$$

(*4*) $$k_{SGS}=\;C_{t}\frac{\mu_{t}}{\rho }S,$$

where, *u, v,* and *w* are the velocity vector components. *u′* denotes the variance in the velocity vector component *u*. *C_t_* is a model coefficient = 3.5 (25), *µ_t_* is the turbulent eddy viscosity, *ρ* is fluid density (1,050 kg/m³), and S is the modulus of the mean strain rate tensor.

Helicity was assessed on cross-sectional planes through the IVC and SVC. Velocities in the entire model were averaged over 1 s of physical time using a sliding window approach with 50 samples to keep the simulation data generated to a manageable size (27 Gb/simulation). Sensitivity of velocity results to the size of the sliding window is reported in Supplemental Table S4. This same method was used to average wall shear stress (WSS) and TKE. To quantify vorticity of the main flow structures simulated in the RA, a volume average of axial-vorticity was calculated. Surface-average WSS was quantified for two surfaces in the RA: the auricle and remaining atrium. This was to assess the thrombogenicity of the auricle region where thrombi have previously been reported and often develop in the left atrium.

### Statistics

All statistics were performed with the Real Statistics Resource Pack (Release 5.7) for Excel (Microsoft, Redmond, WA). Data were tested for normality with a Shapiro–Wilk test. Maximum and minimum diameter variances in the static imaging were compared with a Wilcoxon signed-rank test. Surface-averaged, time-averaged wall shear stress (TAWSS) in the atrium and auricle were compared with a Student’s *t* test. *P* values associated with trendlines were calculated by linear regression.

## RESULTS

Minimum and maximum diameters in the SVC, IVC, RA, and major branching veins varied greatly between subjects (Table 1). Variance in vein diameter measurements, between the four volunteers, was higher for minimum values compared with maximums (mean = 0.33 vs. 0.17, *P* = 0.020).

**Table 1.** Minimum and maximum diameters of the SVC, IVC, RA, and major branching veins for each subject

					Axis Length, cm								Diameter, cm									
					Right Atrium		Inferior Vena Cava		Superior Vena Cava		Left Brach Vein		Right Brach Vein		Left Common Iliac Vein		Right Common Iliac Vein		Left Subclavian Vein		Right Subclavian Vein	
Subject	Sex	Age	Height	Weight	maj	min	max	min	max	min	max	min	max	min	max	min	max	min	max	min	max	min
1	M	64	173	67	3.99	2.03	2.85	0.53	1.84	0.45	1.29	1.19	1.46	0.78	1.98	0.56	1.55	1.05	1.12	0.61	1.50	0.55
2	F	58	170	66	3.24	1.92	3.04	1.38	1.24	0.47	1.39	0.81	1.42	0.43	1.64	1.10	1.94	1.20	1.07	0.37	0.61	0.07
3	F	55	178	76	4.64	1.85	3.30	0.90	2.28	0.73	1.98	1.18	1.39	0.78	2.27	0.96	1.72	0.77	1.29	0.23	1.21	0.47
4	F	56	171	99	5.04	1.73	2.40	0.55	1.61	0.60	2.15	0.63	1.95	1.20	2.29	1.51	2.03	0.98	1.14	0.48	0.82	0.20
Mean					4.23	1.88	2.90	0.84	1.74	0.56	1.70	0.96	1.55	0.80	2.05	1.03	1.81	1.00	1.15	0.42	1.03	0.32
Std. Deviation					0.68	0.11	0.33	0.34	0.38	0.11	0.37	0.24	0.23	0.27	0.26	0.34	0.19	0.15	0.08	0.14	0.34	0.20

brach, brachiocephalic; IVC, inferior vena cava; F, female; M, male; maj, major; max, maximum; min, minimum; RA, right atrium; std, standard; SVC, superior vena cava.

Analysis of the gated computed tomography angiography (CTA) data revealed moderate changes in the maximum and minimum diameters of the SVC and IVC throughout the cardiac cycle (Supplemental Fig. S5). The mean change (maximum diameter − minimum diameter/mean diameter) in the maximum diameter of the SVC and IVC were 18% and 14%, respectively, whereas minimum diameters changed by 33% and 12%, respectively. The RA itself changed most noticeably in major axis length (28%) throughout the cardiac cycle, whereas minor axis length was relatively constant (18%).

Results in the RA were highly sensitive to the extent of the geometry modeled. Average velocity error on a plane through the RA was 30% when comparing *Models A* and *B* and 36% for *Models B* and *C* (Supplemental Fig. S6). Inclusion of the brachial, renal, and iliac veins resulted in highly helical flows entering the RA (Fig. 2). Caval helicity increased with increased model range (476% and 238% in the SVC and IVC, respectively). Imposing a transient inlet waveform to the SVC and IVC influenced the distribution of time-averaged velocities particularly in the IVC, where velocities were underestimated by constant inlet flow (9%). RA velocity distributions were different under transient and constant inlet conditions, with constant inlets slightly underestimating low velocities somewhat, though mean values were nearly equal (3%; Supplemental Fig. S8 and Table S5). Constant inlet conditions were found to have a negligible effect on TAWSS results in the RA when compared with constant boundary conditions (Supplemental Fig. S9 and Table S5). Constant inlet conditions did however lead to an underestimation of TAWSS in the SVC (10%) and IVC (37%; Supplemental Fig. S9 and Table S5). As hematocrit was doubled from 25% to 50%, mean viscosity in the model increased nearly linearly with a 2.1-fold increase and this had no significant impact on flow structures (Supplemental Fig. S4).

**Figure 3.**
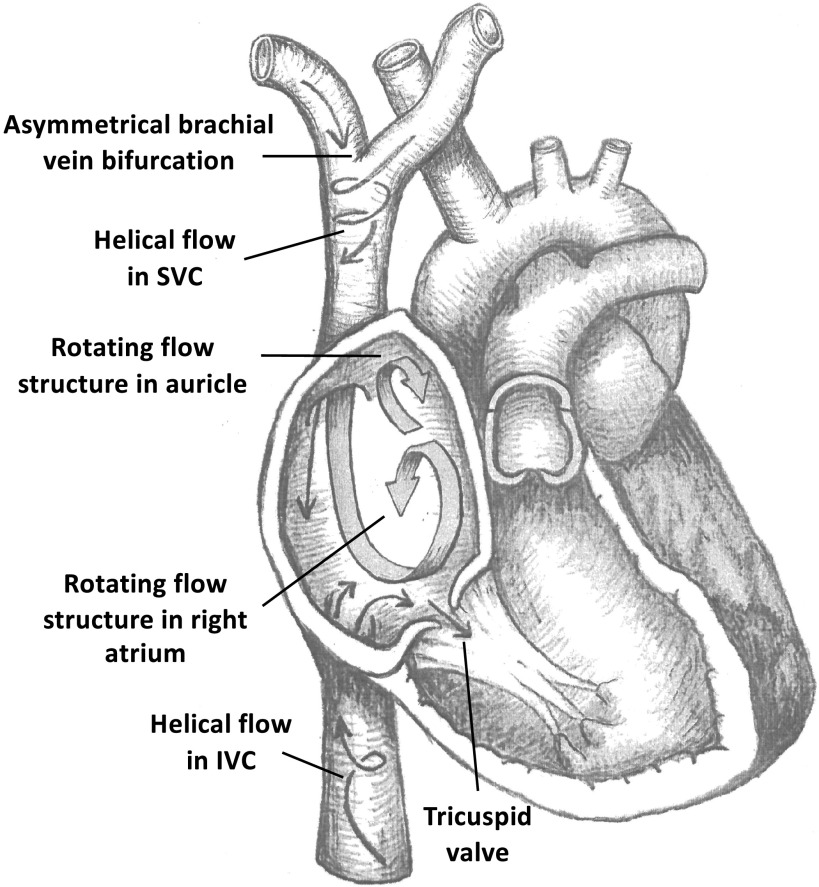
Cross-sectional diagram showing the main flow structures in the superior vena cava (SVC) and inferior vena cava (IVC), and right atrium. Distance between the right atrium and junction of the SVC shortened for visualization purposes.

**Figure 4.**
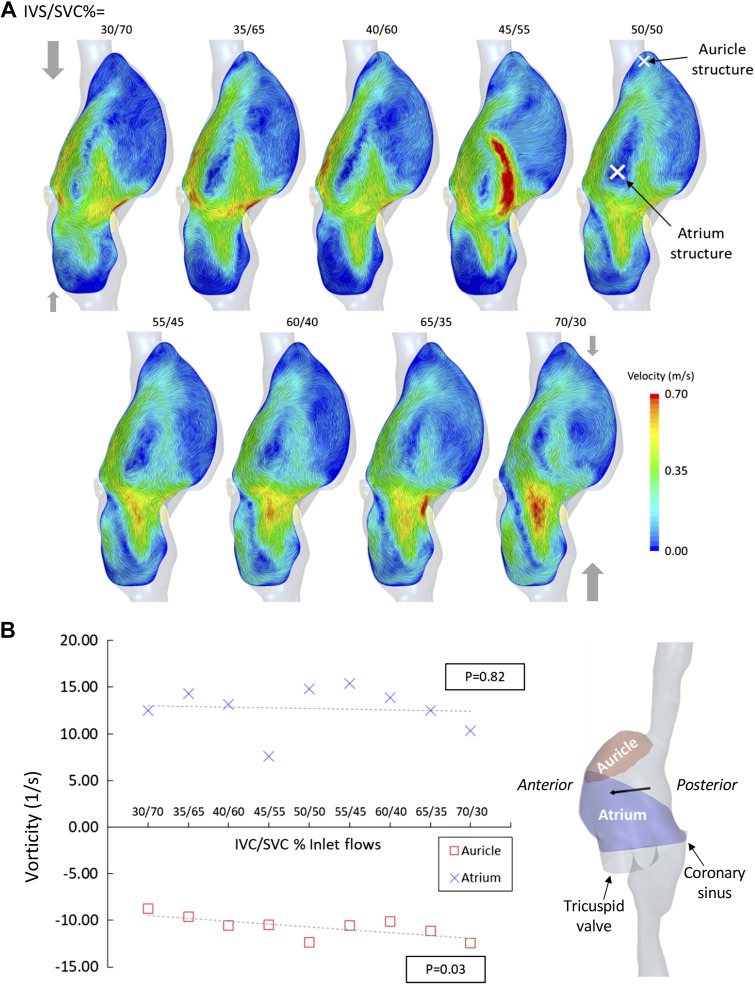
*A*: cross-sectional planes through the center of the right atrium showing a line integral convolution of velocity (anterior view). The centers of the two main rotating flow structures are indicated on the 50/50 flow scenario in white. IVC/SVC indicates the fractional inlet flows (%) assigned to the venae cavae. The gray arrows show the relative flow rates of the venae cavae. *B*: volume-averages of vorticity (the vector component aligned with the vortex core indicated by black arrow) for the rotating flow structures in the auricle and main body of the right atrium (*right*). IVC, inferior vena cava; SVC, superior vena cava.

**Figure 5.**
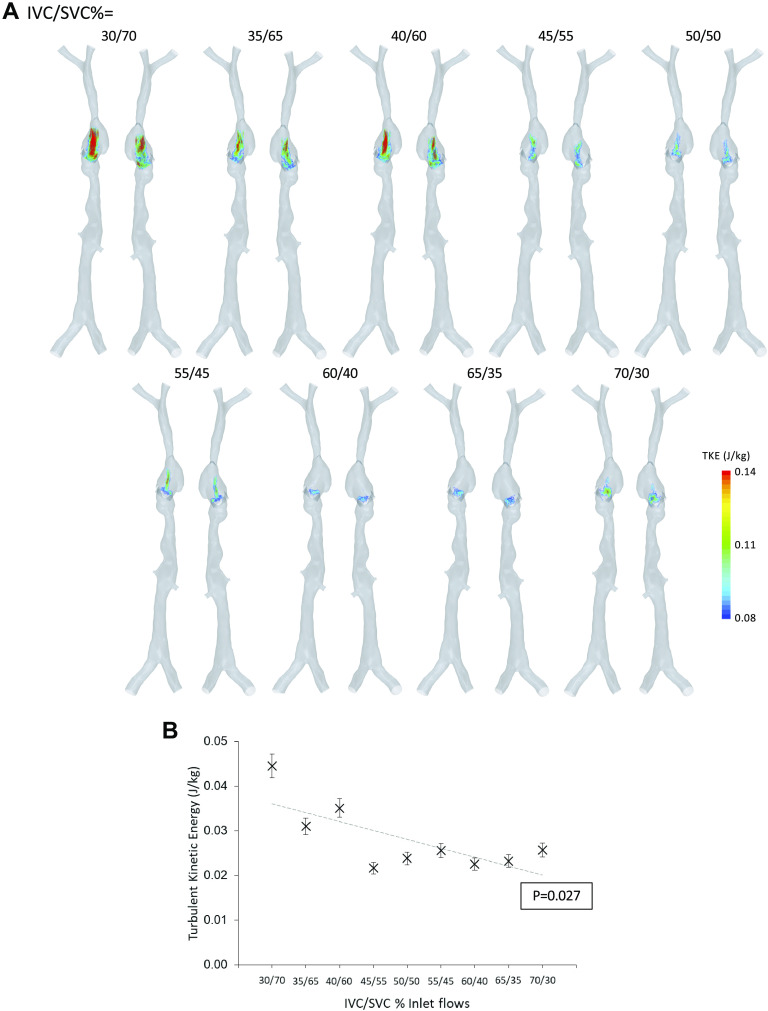
*A*: volume thresholds for turbulent kinetic energy (TKE) >0.08 J/kg shown in posterior (*left*) and anterior (*right*) views for each flow scenario. IVC/SVC indicates the fractional inlet flows (%) assigned to the venae cavae. *B*: volume-averaged TKE in the right atrium plotted for all flow scenarios. IVC, inferior vena cava; SVC, superior vena cava.

**Figure 6.**
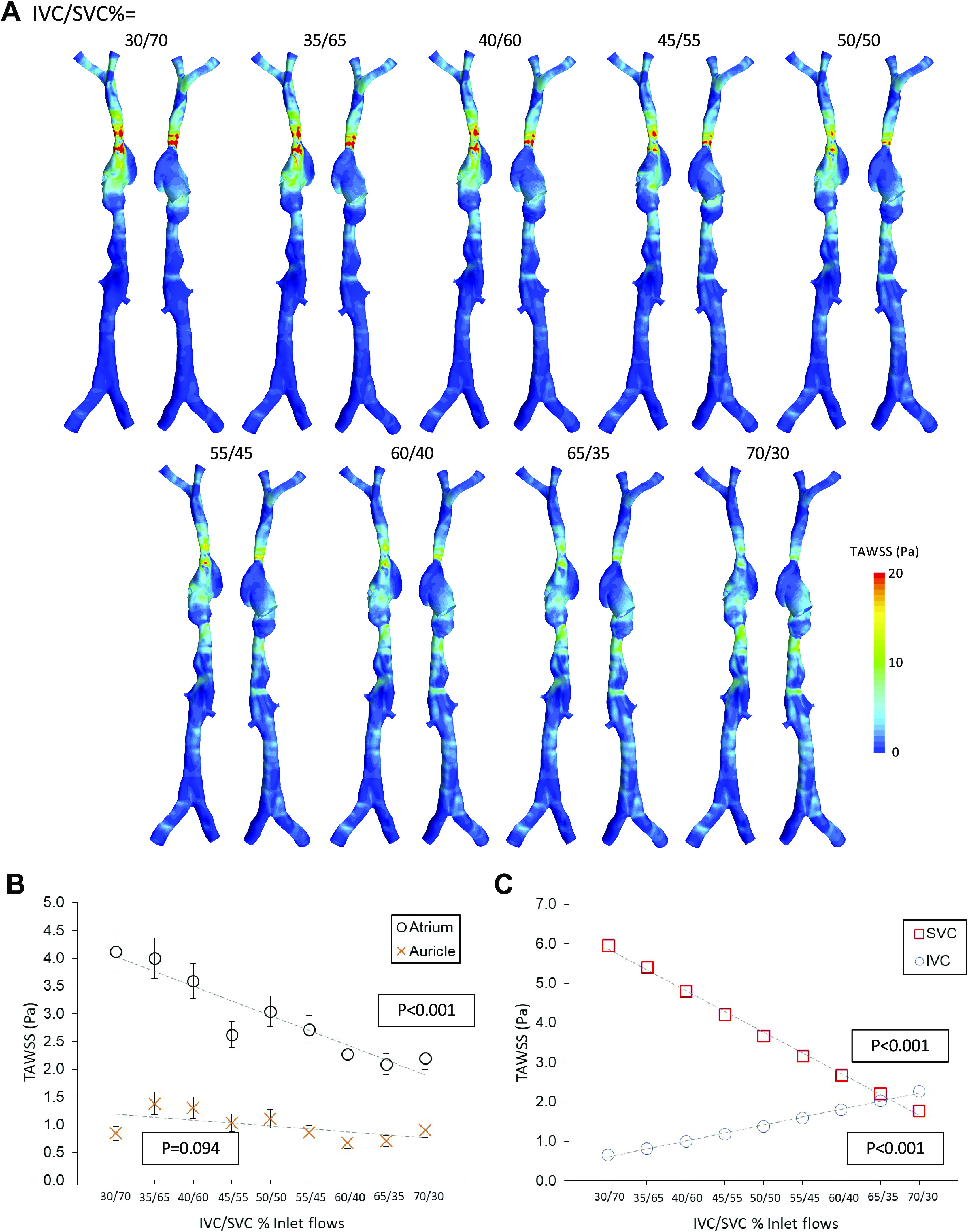
*A*: time-averaged wall shear stress (TAWSS) surface plots, shown in posterior (*left*) and anterior (*right*) views for each flow scenario. Surface-averaged TAWSS in the right atrium and auricle (*B*) as well as the superior vena cava (SVC) and inferior vena cava (IVC; *C*) plotted for all flow scenarios. IVC/SVC indicates the fractional inlet flows (%) assigned to the venae cavae. Graphs *B* and *C* have different *y*-axis scales due to the greater range of SVC TAWSS values.

Time-averaged velocity streamlines show two counter-rotating vortices in the RA, a larger counterclockwise (when viewed from patients right) structure in the main body of the atrium, and another smaller clockwise structure in the auricle (Figs. 3 and 4*A*). Analysis of the volume-averaged vorticity (component aligned with two main vortical flow structures) shows a gradual increase in the vorticity of the auricle structure as IVC flow increases (*P* = 0.03; Fig. 4*B*). No relationship was found between the strength of the larger structure in the atrium and caval flow rates though this structure does appear to be disrupted under the 45/55% (IVC/SVC%) scenario (Fig. 4*B*). Volume-averaged TKE in the RA was reduced significantly as IVC flow increased (*P* = 0.027; Fig. 5, *A* and *B*). TAWSS in the central RA was higher than in the auricle for all flow scenarios (2.0 ± 0.6 Pa, *P* < 0.001; Fig. 6*B*). Central RA TAWSS was reduced with increased IVC flow (*P* < 0.001), whereas no relationship between caval flow and TAWSS was apparent in the auricle (*P* = 0.094; Fig. 6*B*). Surface-averaged TAWSS in the central RA was consistently higher than in the auricle (mean difference = 1.98 ± 0.64 Pa, *P* < 0.001). Vena cava surface-averaged TAWSS also each increased linearly with a greater proportion of total flow (*P* < 0.001), being approximately equivalent in the 65/35% (IVC/SVC%) flow scenario (Fig. 6*C*).

## DISCUSSION

In this study, new insights into RA flow characteristics under a range inlet flow rates from the SVC and IVC are presented. Highly helical flow was established in both the SVC and IVC flows entering the RA. The distal venous morphology appears to be the origin of this helicity. The commonly reported large, rotating structure, combining SVC and IVC flows in the central RA is confirmed here though a previously unreported counter-rotating structure in the right auricle is also present. These flow structures appear across all SVC/IVC flow conditions. RA TKE appears highly dependent on SVC/IVC flow contributions, decreasing as IVC flow increases. As expected, surface-average TAWSS in the venae cavae change with SVC/IVC flow contributions, being approximately equivalent when the IVC delivers 65% of the total flow.

The static imaging data used in this study combine two separate acquisitions, making an extensive venous 3-D reconstruction including the iliac, renal, and brachial veins possible. A high degree of morphological heterogeneity was observed between the four healthy volunteers (Fig. 1), particularly in local minimum diameters. Comparing the morphological data from this study (Table 1) with similar data for the equivalent arteries shows that this heterogeneity is unique to the venous side (26, 27). A likely explanation for this lies in the much lower pressures (5–10 mmHg) (28), thinner vessel wall, and a high degree of compliance of the vena cava. The veins being less rigid can better conform to local anatomy. We can observe this at the SVC junction, where the left brachial vein wraps around the brachiocephalic artery (Fig. 3), creating an asymmetrical junction and imparting high helicity on SVC flow (Fig. 2).

Investigation of model sensitivity to the extent of the reconstruction domain, constant/pulsatile inlet conditions, and hematocrit provide useful information for future CFD studies of the RA. The three models of increasing reconstruction extent (*Models A*, *B*, and *C* in Fig. 2) show the impact of the vena cave, iliac, and brachial veins on RA flow characteristics. We see significant differences in time-averaged velocity in the RA and an appreciable deviation in the orientation of the jet of SVC blood entering the RA (Fig. 2). Analysis of helicity in the caval veins indicates that cropped geometries lack the helicity imparted by branching veins. This changes the nature of the flows entering the RA, impacting the resulting flow structures. These results suggest the importance of distal venous morphology in CFD studies where RA flow structures are of interest. Comparison of constant and transient inlet conditions revealed important spatial differences. Although time-averaged velocity differences were low (<10%) throughout the entire domain, TAWSS showed significant differences in the SVC and IVC (10% and 37%, respectively). For the purposes of this study, only shear results in the RA were of interest, where the difference was low (1%). In future studies where shear stress results in the venae cavae are analyzed, pulsatile inlet conditions would be critical to accurate results. The model was not particularly sensitive to changes in hematocrit, producing very similar flow structures even at the extremes (25% and 50%) for the 50/50 flow scenario. This indicates that perhaps patient-specific hematocrit values are not critical to accurate simulation of RA flow.

Four-dimensional MRI studies provide the most reliable data on RA flow to date. Dewhurst et al. (5) studied 18 patients (21–50 yr) with structurally normal hearts, though nine previously suffered cryptogenic stroke or transient ischemic attack, revealing three distinct flow characteristics in the RA: single vortex (*n* = 13), multiple vortices (*n* = 4), and helical flow (*n* = 1). In the single vortex flow pattern, the SVC and IVC flow contribute to the same vortex in the RA, whereas the multiple vortex cases exhibit two main vortices, one for each caval inflow (5). Parikh et al. (6) conducted a similar study, comparing 13 patients who had suffered cryptogenic stroke or transient ischemic attack (21–50 yr) with 13 control subjects (25–50 yr). In the control group, a clockwise vortical structure composed of SVC and IVC flow was most common in both systole (*n* = 8) and diastole (*n* = 10), whereas alternative flow characteristics, similar to those described by Dewhurst et al. (5) (multiple vortices, helical flow), were observed more frequently in patients with a history of stroke or ischemic attack (systole: *n* = 11, diastole: *n* = 7) (6). Parikh et al. (6) concluded that the structurally normal heart can exhibit a range of flow characteristics in the RA and that this is a result of unique caval vein arrangements. The dominant flow pattern simulated in the present study (Figs. 3 and 4*A*), seen to differing degrees across all flow scenarios, is that of a large counterclockwise vortex in the main body of the atrium. This is generated when SVC flow encounters incoming IVC flow and is turned back on itself carrying some IVC flow with it. A counter-rotating vortex then results in the right auricle. Vorticity magnitude has been reported previously to quantify the rotational nature of the flow in the RA, though no distinction was made between the flow structures that direct blood toward the tricuspid valve and counterproductive vortical structures (29). Here, we take a common vector component (Fig. 4*B*) of vorticity, aligned with the two prominent flow structures. The strength of the central vortical structure shows no relationship with IVC/SVC flow fractions, although the structure appears somewhat disrupted under the 45/55 flow scenario. There is a mild increase in the strength of the auricle vortex as IVC flow dominates potentially reducing flow stasis. These results reflect the commonly observed single vortex pattern in the body of the atrium, though generally this is described as a clockwise rotation (5, 10). The same counterclockwise structure has however been reported in previous CFD (29, 30) and imaging studies, where it was more prevalent (20.5%) in older patients (60–80 yr). Why the smaller auricle structure has not been reported in previous 4-D-MRI studies is likely due to the comparatively low spatial resolution of 4-D-MRI. Current 4-D-MRI postprocessing produces a flow field with a resolution of 1–3 mm³ (5, 6), whereas the computational mesh used in the present study has a mean resolution in the RA of 0.02 mm³. It is likely that such structures are not visible on the coarser grid of velocities provided by 4-D-MRI.

High helicity in the vena cava, particularly in the IVC, has been observed with 4-D-MRI (5). However, the limited imaging domain (240 mm inferior-superior) in the study meant that the origin of this helicity was unclear. Past CFD (29, 30) and benchtop experiments (31) in the RA have been based on similar imaging domains requiring truncation of the SVC and IVC in reconstructions and these have not made mention of such helicity. Helical flow in both vena cava was again visible in the results of the present study. These results demonstrate the necessity of reconstructing an extensive venous model, which includes the brachial and iliac arteries to capture caval helicity and the resulting effect on RA flow characteristics (Fig. 2). Truncation of the IVC and SVC may be one reason that the smaller vortex in the auricle has not been reported in previous CFD or benchtop studies.

A novel aspect of the current study is the application of an LES computational scheme allowing us to resolve large turbulent flow structures while accounting for the influence of smaller structures (smaller than the computational grid). An LES scheme was implemented over a less computationally expensive RANS scheme because early simulations indicated that the complex and transient vortex structures of the RA were not well characterized by this approach. Previous CFD investigations in the RA have applied a laminar flow assumption justified by simple Reynolds number calculations (29). This assumption is not appropriate for the present study where spectral analysis confirms the presence of turbulent flow throughout the RA (Supplemental Fig. S10).

TKE is the kinetic energy associated with eddies in turbulent flow. This energy is ultimately transferred into increasingly smaller eddies eventually being dissipated by viscous forces. Calculation of TKE in the RA is applied in the present study to quantify the efficiency of RA mixing across flow scenarios. The same approach has been applied previously in the right ventricle (RV) to assess outcomes from congenital heart disease repair, observing similar average RV TKE (1.24 ± 0.53 mJ) in a control group (*n* = 10) to average RA TKE in the present study (1.33 ± 0.34 mJ; Fig. 5*B*) (32). Though the relationship between TKE and heart function is yet to be clearly defined, it is expected that an increase in TKE in the RA would lead to additional energy losses requiring extra work by the muscles of the heart, for the same cardiac output. The results from the current study suggest that increased SVC flow elevates TKE significantly in the RA. Increasing the flow entering from the SVC, as is common in VV ECMO (blood being drained from the IVC and returned to the SVC), will likely increase energy losses through turbulence. Beyond less efficient heart function, high TKE might impact ECMO and dialysis through increased thrombosis and hemolysis. Turbulent flow is often associated with increased endothelial cell misalignment, retraction, and loss (33), increasing susceptibility to thrombosis. Patients with atrial fibrillation displaying turbulent left atrium flow are at increased risk of thromboembolic events (34) and the turbulent conditions in the wake of atherosclerotic plaques in the coronary arteries are known to promote thrombosis (35). Hemolysis is the rupturing of red blood cells and often occurs under flow-induced stresses, in particular turbulent stresses (36, 37). Pediatric patients on ECMO with severe hemolysis have been observed to have a sixfold increased risk of in-hospital mortality and increased plasma-free hemoglobin has been associated with greater mortality in adult patients on ECMO (38). Similarly, hemolysis is recognized as a complication of hemodialysis (39). Taken together, the literature suggests that increased TKE would have a detrimental effect on patients on ECMO and dialysis. Comparing patient outcomes with direct measurement of TKE in vivo or patient-specific CFD investigations could provide data to support this hypothesis. As the spatial resolution of 4-D-MRI improves or if CFD analysis is used clinically, TKE may become a useful indicator of RA function and susceptibility to thrombotic events.

The resolution of 4-D-MRI also has implications for the calculation of WSS, making it only possible to estimate the near-wall shear rate of blood (40), generally resulting in an underestimation (41). CFD provides more near-wall data points improving WSS estimation, in an equivalent flow field. Data from this study show a linear increase in central RA TAWSS with greater SVC flow (Fig. 6*B*). This is most likely a result of the orientation of the caval veins, where the SVC inflow follows the RA wall more closely than the IVC inflow. As expected, TAWSS in the caval veins exhibits a linear relationship with inlet flows (Fig. 6*C*) with greater inflow yielding higher TAWSS. Due to the larger diameter of the IVC, equilibrium in surface-averaged TAWSS was established at the flow conditions expected in healthy adults (IVC/SVC = 65/35%) (20). TAWSS in the auricle was on average 2 Pa lower than in the rest of the atrium (*P* < 0.001). Stasis (low TAWSS) in the left atrium is common in patients with atrial fibrillation (42) and the left auricle is known to be thrombogenic under such conditions (43). Though much rarer, thrombosis in the right auricle has also been observed (44). It, therefore, is feasible that turbulent RA flow may provoke thrombosis in the right auricle, though this hypothesis requires investigation in a longitudinal study.

These results provide a basis for the optimization of VV ECMO and hemodialysis via central dialysis catheter. Analysis of TKE in the RA suggests that optimal mixing occurs when IVC flow accounts for majority of the inflow. In addition to this, when IVC flow is high (65% of total flow), shear stresses in the vena cava are approximately equal and deformation of the veins is likely to be minimal. These results suggest that taking a cannulation approach that ensures greater IVC inflow may improve treatment effectiveness. These results may also increase understanding of different clinical situations and serve as a basis for further investigations concerning recirculation in VV ECMO and hemodialysis.

Several limitations of this study should be outlined. A patient-average model was constructed based on a small group of healthy volunteers. Although the model was modified to represent the average diameters and distance between landmark points of the group, it is still comprised patient-specific components. A larger group of patient-specific simulations is required to give more generalized data. As in previous CFD studies, the atrium was modeled as rigid (29, 30, 45, 46). We know from basic physiology and the transient imaging results in this study (Supplemental Fig. S5) that the RA contracts during the cardiac cycle. Similarly, the tricuspid valve was simulated as rigid and fully open, as in previous studies (29, 30, 45, 46). The vena cava is omitted from previous CFD studies of the RA. Even though included in this study, the venae cavae too are assumed to be rigid in our simulations but are compliant in reality. Taken together, the assumption of rigid walls is likely to have an impact on the flow patterns simulated. Data are scarce on the validation of such an approach. However, Wong et al. (46) reported good agreement in transient flow structures (quantified by vorticity contours) between a rigid-wall CFD model and phase-contrast MRI of the RA. The rigid-wall assumption has also been compared with in vivo 4-D-MRI data for a similar simulation of total cavo-pulmonary connection, showing reasonable agreement (47). These results suggest that the rigid-wall model, as applied here, should provide a reasonable approximation of prevailing flow structures, though further validation would provide important data regarding sensitivity. A fluid structure interaction (FSI) model is a potential solution to include motion though this requires considerably more computational resources and would introduce further sensitivity due to assumptions about vessel wall material properties. A common vector component of vorticity was taken to quantify the strength of the main RA flow structures (Fig. 4*B*). This vector is an estimation of the mean orientation of these structures across all flow scenarios and the results are sensitive to small changes in direction. The flow rate into the system was set to a constant 6 L/min. Doppler velocity data to estimate actual flow waveforms were not available for this group, though the effect of constant inlets was shown to have minimal impact on results. Though the RA flow structures presented here show good agreement with those reported previously in imaging studies, they could not be directly compared with imaging observations from the volunteers as such data were not available. In future studies, such models would benefit from validation against benchtop measurements or in the case of patient-specific geometries, velocity measurements obtained from imaging. Investigating mesh sensitivity showed that although velocity results were well converged, TAWSS and, to a lesser degree, TKE still exhibited some differences when compared with a mesh of approximately double the cell count (Supplemental Table S3). The error that this introduces is shown in Figs. 5*B* and 6*B*. For the purposes of the present study where surface and volume averages are compared across cases, and reported differences are sufficiently large, these errors are acceptable. In future studies where localized TAWSS and TKE quantities are to be compared, greater temporal and/or spatial resolution should be used to achieve a greater degree of mesh convergence for these quantities.

### Conclusions

The authors present a fully resolved CFD model for the complete venae cavae and RA, including the coronary sinus. A range of IVC and SVC flow rates were simulated and the commonly reported large vortex structure in the central RA is confirmed here for all inlet conditions. An additional smaller, counter-rotating vortex was also visible in the auricle. Caval arrangement and RA geometry appear to induce this rotational mixing and minimize TKE when IVC flow dominates, as is the norm in healthy adults. The extensive geometry including the brachial and iliac vein junctions appear critical for capturing the helicity of the flows entering the RA and accurately modeling the major flow structures. WSS is consistently low in the auricle compared with the rest of the atrium. Analysis of model sensitivities suggests that distal venous morphology is crucial to accurate characterization of RA flow. Though not the focus of the present study, if TAWSS in the venae cavae is of particular interest, pulsatile boundary conditions should be applied. The model was not particularly sensitive to changes in hematocrit.

## SUPPLEMENTAL DATA

Supplemental Figs. S1–S10 and Supplemental Tables S1–S5: https://doi.org/10.6084/m9.figshare.16602209.

## GRANTS

The computations were enabled by resources provided by the Swedish National Infrastructure for Computing (SNIC) at the High-Performance Computing Center North partially funded by the Swedish Research Council through Grant Agreement No. 2016–07213. The authors also acknowledge Partnership for Advanced Computing in Europe (PRACE) for awarding us access to the Supercomputer Mahti at the CSC IT Center for Science, Finland (Grant number: 17DECI0055). This work was supported by Region Stockholm through Grant No. HMT2018.

## DISCLOSURES

L.M.B. is a member of the Medical Advisory Boards of Eurosets Srl., Medolla, Italy, and Xenios AG, Heilbronn, Germany. No devices however are simulated or discussed in this study. The focus is RA physiology. None of the other authors has any conflicts of interest, financial or otherwise, to disclose. 

## AUTHOR CONTRIBUTIONS

L.P.P., A.S.M., T.B.B., L.M.B., and L.P.W. conceived and designed research; L.P.P., A.S.M., and T.B.B. performed experiments; L.P.P. analyzed data; L.P.P., L.M.B., and L.P.W. interpreted results of experiments; L.P.P. prepared figures; L.P.P. drafted manuscript; L.P.P., A.S.M., T.B.B., L.M.B., and L.P.W. edited and revised manuscript; L.P.P., A.S.M., T.B.B., L.M.B., and L.P.W. approved final version of manuscript. 
